# Priming With Recombinant BCG Expressing Novel HIV-1 Conserved Mosaic Immunogens and Boosting With Recombinant ChAdOx1 Is Safe, Stable, and Elicits HIV-1-Specific T-Cell Responses in BALB/c Mice

**DOI:** 10.3389/fimmu.2019.00923

**Published:** 2019-05-14

**Authors:** Athina Kilpeläinen, Narcís Saubi, Núria Guitart, Nathifa Moyo, Edmund G. Wee, Krupa Ravi, Tomáš Hanke, Joan Joseph

**Affiliations:** ^1^AIDS Research Unit, Infectious Diseases Department, Catalan Center for HIV Vaccine Research and Development, Hospital Clínic/IDIBAPS, Barcelona, Spain; ^2^The Jenner Institute Laboratories, University of Oxford, Oxford, United Kingdom; ^3^International Research Center for Medical Sciences, Kumamoto University, Kumamoto, Japan

**Keywords:** mycobacteria, HIV, conserved, vaccine, rBCG, live-attenuated vaccines

## Abstract

BCG is currently the only licensed vaccine against tuberculosis (TB) and confers protection against meningitis and miliary tuberculosis in infants, although pulmonary disease protection in adults is inconsistent. Recently, promising HIV-1 immunogens were developed, such as the T-cell immunogens “tHIVconsvX,” designed using functionally conserved protein regions across group M strains, with mosaic immunogens to improve HIV-1 variant match and response breadth. In this study, we constructed an integrative *E. coli*-mycobacterial shuttle plasmid, p2auxo.HIVconsvX^int^, expressing the immunogens HIVconsv1&2. This expression vector used an antibiotic resistance-free mechanism for plasmid selection and maintenance. It was first transformed into a glycine auxotrophic *E. coli* strain and subsequently transformed into a lysine auxotrophic *Mycobacterium bovis* BCG strain to generate vaccines BCG.HIVconsv1^2auxo.int^ and BCG.HIVconsv2^2auxo.int^. The DNA sequence coding for the HIVconsv1&2 immunogens and protein expression were confirmed and working vaccine stocks were genetically and phenotypically characterized. We demonstrated that BCG.HIVconsv1&2^2auxo.int^ in combination with ChAdOx1.tHIVconsv5&6 were well tolerated and induced HIV-1-specific T-cell responses in adult BALB/c mice. In addition, we showed that the BCG.HIVconsv1&2^2auxo.int^ vaccine strains were stable *in vitro* after 35 bacterial generations and *in vivo* 7 weeks after inoculation. The use of integrative expression vectors and novel HIV-1 immunogens are likely to have improved the mycobacterial vaccine stability and specific immunogenicity and may enable the development of a useful vaccine platform for priming protective responses against HIV-1/TB and other prevalent pediatric pathogens shortly following birth.

## Introduction

There is an urgent need for the development of a safe, effective and accessible vaccine against HIV-1. Although highly active antiretroviral therapy has made HIV-1 a chronic and manageable infection, the disease continues to be a huge burden on healthcare systems. This is the case especially in resource-poor settings such as Sub-Saharan Africa, where only 60% of infected individuals were receiving treatment in 2017 (1). For neonates, children and adults, a preventive vaccine would be the best solution for both infection prevention and reduction of HIV-1-related morbidity. An estimated 1,000,000 people living with HIV-1 became co-infected with *Mycobacterium tuberculosis* (*Mtb*) in 2017, and 300,000 people were estimated to have died from tuberculosis (TB) (2). The current licensed vaccine, *M. bovis* bacillus Calmette–Guérin (BCG) which protects against childhood TB meningitis, milliary TB disease, and leprosy, is currently administered to 80% of infants in countries where it is part of the national childhood immunization programme (3). The development of a combined vaccine, which would confer protection against TB and mother-to-child transmission (MTCT) of HIV-1 through breastfeeding in neonates, would be a useful tool in the fight against both global diseases.

BCG has several advantages as a vaccine vector: it is (i) easy to mass-produce with low cost, (ii) heat stable (4), (iii) suitable for neonates as vaccination is not affected by maternal antibodies (5, 6), and (iv) it acts as an adjuvant on its own due to its cell-wall components and lipoproteins (7–10). BCG is estimated to have been used in over 3 billion individuals and thus has a long and demonstrated safety record (11). Although BCG is not recommended in HIV-1-infected individuals, the latest position of the World Health Organization is that infants of unknown HIV-1 status are to be vaccinated, should they have no clinical evidence suggestive of HIV-1 infection. In addition, BCG vaccination can be administered after starting anti-retroviral therapy (ART) and once the infant is confirmed to be immunologically stable (CD4 >25%) (12).

There is a growing body of evidence demonstrating that T-cell responses directed against HIV-1 play a role in controlling viral replication (13–17). For example, HIV-1-specific CD8^+^ T-cells were detected in exposed seronegative individuals (13). BCG is a promising live attenuated bacterial vaccine vector for inducing T-cell immunity. It was shown that BCG expressing HIV-1/SIV antigens induced transgene product-specific T-cell responses in several animal models (6). The combination of rBCG and a replication-deficient vaccinia virus strain expressing SIVgag was shown to protect cynomolgus macaques against mucosal challenge with pathogenic SIV (18). Furthermore, administration of HIV-1 vaccine candidates is required in remote areas of developing countries with a high burden of HIV-1 and TB infection, a successful vaccine will have to be heat-stable, safe, cheap, and easy to mass produce.

Our groups have been working for a long time on recombinant BCG based HIV vaccine development, assessing prime-boost vaccination regimens, the influence of age and immunization routes, and engineering a new mycobacterial vaccine design by using an antibiotic-free plasmid selection system (19–21). We, recently developed the integrative *E. coli*-mycobacterial shuttle plasmid p2auxo.HIVA^int^ expressing the HIV-1 clade A immunogen HIVA (22). The shuttle vector uses an antibiotic resistance-free plasmid selection and maintenance system, using glycine complementation for expression in *E. coli* and lysine complementation in mycobacteria (23). The prototype BCG-vectored HIV-1 vaccine candidate, BCG.HIVA^2auxo.int^, was safe and induced HIV-1- and *M. tuberculosis*-specific interferon-γ-producing T-cell responses when administered as part of a prime-boost immunization regimen with modified vaccinia Ankara-vectored MVA.HIVA in adult BALB/c mice (23).

In this study, we constructed novel BCG-vectored HIV-1 vaccine candidates expressing immunogens HIVconsv1 and HIVconsv2. These are the 2nd-generation conserved-region immunogens aiming to induce effective T-cell responses against conserved regions of the HIV-1 proteome, which are common to most global HIV-1 variants and in which mutations often lead to loss of replicative fitness (24). Prime-boost regimens delivering the 1st-generation conserved immunogen HIVconsv, combining DNA, simian adenovirus, and MVA were safe and immunogenic in 8 clinical trials in both HIV-1-positive individuals on ART and uninfected humans. This supported the concept by inducing human effector T cells capable of recognizing virus-infected autologous CD4^+^ cells and inhibiting HIV-1 replication of 4 major clades *in vitro* (25–27). The 2nd-generation HIVconsvX immunogens were designed by redefining the group M conserved regions, with the assistance of bioinformatics to include epitopes identified in untreated HIV-1 infected patients with low viral loads, and utilizes a bi-valent mosaic design to maximize the match of potential 9-mer T-cell epitopes in the vaccine to global variants (28). Here we describe the construction and characterization of recombinant BCG expressing the HIVconsv1 and HIVconsv2 immunogens, the BCG.HIVconsv1^2auxo.int^ and BCG.HIVconsv2^2auxo.int^ vaccines, harboring the integrated 2auxo expression cassette in their chromosome. In particular, we demonstrated that BCG.HIVconsv1&2^2auxo.int^ in combination with ChAdOx1.tHIVconsv5&6 were well tolerated and induced HIV-1-specific T-cell responses in adult BALB/c mice. In addition, we showed that BCG.HIVconsv1&2^2auxo.int^ vaccine strains were stable *in vitro* and *in vivo*. The utilization of the new BCG-vectored vaccine platform is discussed.

## Materials and Methods

### Construction of the BCG.HIVconsv1&2^2auxo.int^ Strains Using an Antibiotic-Free Plasmid Selection System

The double auxotrophic *E. coli*–mycobacterial shuttle integrative vector, the p2auxo.int plasmid, was previously constructed in our laboratory (23). This vector contains the *glyA* and l*ysA* genes which function as an antibiotic-free selection system in the auxotrophic strains of *E. coli* M15Δ*glyA* and BCG ΔLys, respectively. The synthetic sequences of HIVconsv1 and HIVconsv2 (28) were codon optimized for BCG expression and synthesized by Geneart (USA). The genes were ligated to the integrative p2auxo.int plasmid fused to the 19-kDa lipoprotein secretion signal sequence generating p2auxo.HIVconsv1^int^ and p2auxo.HIVconsv2^int^ containing sites for integration into the BCG genome at the attB site. The plasmids were subsequently transformed into the *E. coli* M15Δ*glyA* strain.

### Bacterial Cultures and Transformation

Cells of the glycine auxotrophic strain of *E. coli*, M15ΔGlyA (Invitrogen), provided by Dr. Pau Ferrer, were cultured in minimal M9-derivative medium (M9-D: Na_2_HPO_4_, 6.78 g/l; KH_2_PO_4_, 3 g/l; NaCl, 0.5 g/l; NH_4_Cl, 1 g/l, glucose, 10 g/l; MgSO_4_, 2 mmol/l; CaCl_2_, 0.1 mmol/l; thiamine, 0.1 g/l; FeCl_3_, 0.025 g/l; AlCl_3_·6H_2_O, 0.13 mg/l; ZnSO_4_·7H_2_O, 2.6 mg/l; CoCl_2_·6H_2_O, 0.47 mg/l; CuSO_4_·H_2_O, 4.6 mg/l; H_3_BO_3_, 0.03 mg/l; MnCl_2_·4H_2_O, 4.2 mg/l; NiCl_2_·6H_2_O, 0.02 mg/l; Na_2_MoO_4_·2H_2_O, 0.06 mg/l), supplemented with glycine (70 μg/ml). The *E. coli* M15ΔGly cells were transformed with the p2auxo.HIVconsv1^int^ and p2auxo.HIVconsv2^int^ plasmids by electroporation. For this, the *E. coli* cultures were grown to an optical density of 0.9 at 600 nm, transformed using a Bio-Rad gene pulser electroporator at 2.5 kV, 25 μF, and 200 Ω. The transformed cells were subsequently cultured on M9-D agar plates (components as previously described, with 1.5% bactoagar added) without glycine supplementation for selection or with glycine supplementation as a control.

The lysine auxotrophic BCG strain, BCGΔlys, kindly provided by W.R. Jacobs Jr., B.R. Bloom, and T. Hsu was transformed with p2auxo.HIVconsv1^int^ and p2auxo.HIVconsv2^int^ plasmids by electroporation. The mycobacteria were cultured in Middlebrook 7H9 broth medium or on Middlebrook agar 7H10 medium supplemented with albumin-dextrose-catalase (ADC; Difco) containing 0.05% Tween 80. L-lysine monohydrochloride (Sigma) was dissolved in distilled water and used as a supplement at a final concentration of 40 μg/ml. For transformation, BCG was cultured to an optical density of 1.5 at 600 nm, transformed using a Bio-Rad gene pulser electroporator at 2.5 kV, 25 μF, and 1,000 Ω. The transformants were then cultured on ADC-supplemented Middlebrook agar 7H10 medium containing 0.05% Tween 80 without lysine supplementation. The commercial BCG Danish 1,331 strain (Pfizer), was kindly provided by Dr. Neus Altet and the commercial BCG Connaught strain (ImmuCyst, Aventis), from the Urology Department at Hospital Clínic de Barcelona.

### Sodium Dodecyl Sulfate–Polyacrylamide Gel Electrophoresis and Western Blot Analysis

Cell lysates of mid-logarithmic-phase BCG transformants were prepared by sonication in a protein extraction buffer (50 mmol/l Tris–HCl pH 7.5, 5 mmol/l EDTA, 0.6% sodium dodecyl sulfate) containing protease inhibitor cocktail (Sigma). They were subsequently separated by sodium dodecyl sulfate–PAGE, and electroblotted onto a pretreated polyvinylidene difluoride membrane. The HIVconsv1 and HIVconsv2 proteins were stained using the primary anti-Gag monoclonal antibody (38:9k) kindly provided by Dr. Britta Wahren and Jorma Hinkula followed by secondary Rabbit anti-mouse IgG-HRP antibody (Sigma). The membrane was developed using an enhanced chemiluminescence kit (Pierce, Rockford, IL) and visualized using the ImageQuant LAS500 gel imaging system (GE Healthcare, Piscataway, NJ).

### *In vitro* Stability of the BCG.HIVconsv1&2^2auxo.int^ Strains

Five subcultures (~35 bacterial generations) of BCG.HIVconsv1&2^2auxo.int^ (working vaccine stocks) harboring the p2auxo.HIVconsv1&2^int^ plasmid DNAs containing the lysine complementing gene were cultured in 7H9 broth with and without selection (L-lysine for Δ*lysA* strains). Subcultures were performed every 7 days, by transferring 100 μl of the stationary phase culture to 5 ml of fresh medium. PCR analysis of the HIVconsv1&2 DNA coding sequences were performed.

### *In vivo* Stability of the p2auxo.HIVconsv1&2^int^ Plasmids

Growth of BCG.HIVconsv1&2^2auxo.int^ as well as the *in vivo* stability of the integrative p2auxo.HIVconsvX^2auxo^ plasmids were confirmed by recovering of BCG.HIVconsv1&2^2auxo.int^ colonies from the spleens of vaccinated mice 7 weeks after immunization with 10^6^ cfu of BCG.HIVconsv1&2^2auxo.int^ (i.d.) and 10^8^ IU of ChAdOx1.tHIVconsv5&6 (i.m.). Spleens were homogenized and plated onto Middlebrook 7H10 agar plates, and the resulting BCG colonies were cultured in liquid 7H9 medium. Mycobacterial DNA was isolated and PCR analysis was performed using specific primers directed toward both the HIVconsv1 and HIVconsv2 coding sequences in all colonies.

### Sample Preparation for the Multiplex PCR Assay and for attR and attL DNA Regions PCR

For isolation of DNA from wild-type BCG, BCG.HIVconsv1&2^2auxo.int^; 2 ml of mycobacterial culture was centrifuged at 5,000 rpm for 10 min at room temperature. The pellet was resuspended in 200 μl of distilled water and heated at 95 °C for 20 min to inactivate and lyse bacterial cells. The sample was next centrifuged at a speed of 13,000 x*g*. 5 μl of supernatant was used for the amplification reaction or stored at −20 °C. The commercial BCG strains were treated similarly, except in this case, 400 μl of the reconstituted freeze-dried flasks were used.

### Multiplex PCR Assay for *M. bovis* BCG Substrain Pasteur Identification

The multiplex PCR assay was performed as described previously by Bedwell et al. (29), using 5 μl of mycobacterial DNA isolated from BCG.HIVconsv1&2^2auxo.int^, Pasteur, and commercial BCG strains as template in a final reaction volume of 50 μl.

### *E. coli* Plasmid DNA Extraction

The QIAprep Spin Miniprep Kit was used according to the manufacturer's instructions (Qiagen, Hilden, Germany) to extract plasmid DNA from *E. coli*.

### Immunization of Mice and Isolation of Splenocytes

Groups of 16 adult (7-week-old) female BALB/c mice were immunized with 10^6^ CFU of BCG in the footpad. The first group received a 1:1 mixture of BCG.HIVconsv1&2^2auxo.int^, the second group received BCG wt, and two groups were left unimmunized. After 5 or 12 weeks, the mice were boosted with 10^8^ IU of ChAdOx1.tHIVconsv5&6 delivered intramuscularly as a mixture except for one group which was left unimmunized. Mice were sacrificed 2 weeks after the boost for endpoint immunological assays. Immunological mouse experiments were approved by the local Research Ethics Committee (Procedure Med 365/16, Clinical Medicine, School of Medicine, and University of Barcelona) and by the Ethical Committee for animal experimentation from the University of Barcelona, and they strictly conformed to the Generalitat de Catalunya animal welfare legislation.

Groups of five 6-week old female BALB/c were intradermally immunized with 10^6^ CFU of BCG. The first group received a 1:1 mixture of BCG.HIVconsv1&2^2auxo.int^, the second group received BCG wt, the third group received a 1:1 mixture of BCG.HIVconsv1&2^2auxo.int^ intradermally, and one group was left unimmunized. After 5 weeks, the fourth group of mice was boosted with 5 × 10^6^ plaque forming units (PFU) of MVA.tHIVconsv3&4 delivered intramuscularly as a mixture. Mice were sacrificed 1 week later the boost for endpoint immunological assays. All procedures and care were approved by the local Clinical Medicine Ethical Review Committee, University of Oxford and conformed strictly to the United Kingdom Home Office Guidelines under the Animals (Scientific Procedures) Act 1986. Experiments were conducted under project license 30/3387 held by TH.

Immediately following sacrificing of the animals, splenocytes were harvested and homogenized using cell strainers (Falcon; Becton Dickinson) and 5-ml syringe rubber plungers. Red blood cells were removed with ACK lysing buffer (Lonza, Barcelona, Spain), and the splenocytes were washed and resuspended in complete medium (R10 (RPMI 1640 supplemented with 10% fetal calf serum and penicillin–streptomycin), 20 mmol/l HEPES, and 15 mmol/l 2-mercaptoethanol).

### Intracellular Cytokine Staining

For assessing the immunogenicity of the HIVconsv proteins in the BALB/c mice, two HIV-1 peptides corresponding to the amino acid sequences of HIVconsv1 and HIVconsv2, respectively, were used. The following HIV-1 Gag peptides were used: AMQMLKDTI and AMQMLKETI, and HIV-1 protease peptides; VLVGPTPVNI and VLIGPTPVNI. Staining was performed in a 96-well round-bottom plate (Costar, Corning, NY). One million splenocytes were added per well and stimulated with 2 μg/ml of each peptide for 60 min at 37 °C with 5% CO_2_. GolgiStop (Becton Dickinson) containing monensin was then added and the cells were incubated for 5 h. The reaction was then stopped by cooling to 4 °C. The cells were washed with wash buffer (phosphate-buffered saline, 2% fetal calf serum, 0.01% azide) and blocked with anti-CD16/32 (BD Biosciences) at 4 °C for 30 min. The following antibody staining procedures were performed under the same conditions. Cells were washed and stained with anti-CD8-PerCP (BD Biosciences) and anti-CD107a-FITC, washed again, and permeabilized using the Cytofix/Cytoperm kit (BD Biosciences). Cells were washed with Perm/wash buffer (BD Biosciences) before staining with anti-IFN-γ-APC and anti–tumor necrosis factor-α-PE (BD Biosciences). Cells were fixed with CellFIX (BD) and stored at 4 °C until analysis. All chromogen-labeled cells were analyzed in a Becton Dickinson FACScalibur, using the CellQuest software for acquisition (Becton Dickinson) and the Flow-Jo software (Tree Star, Ashland, OR) for analysis.

### Statistical Analysis

Immunogenicity and body mass data are shown as group means with error bars indicating standard deviation. Body mass data are depicted as group means, as well as means ± 2 standard deviation (SD) from naïve mice. Statistical significance was determined by one-way analysis of variance for cumulative frequencies of cytokine producing cells or 2-way analysis of variance for individual cytokine parameters. GraphPad Prism 6.0 software was used.

## Results

### Construction of the BCG.HIVconsv1^2auxo.int^ and BCG.HIVconsv2^2auxo.int^ Vaccine Strains

**Figure 1:**
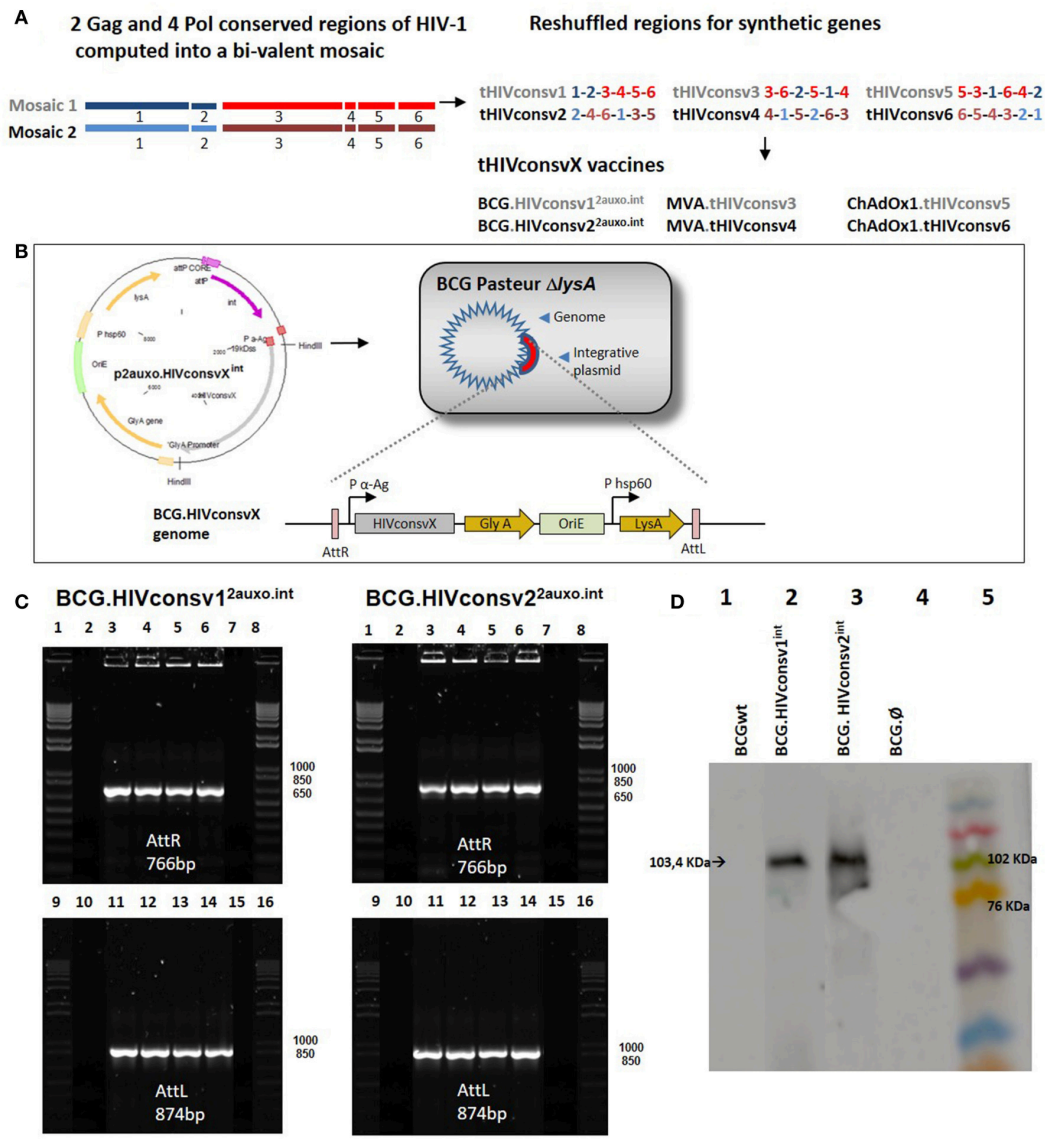
Construction of the BCG.HIVconsv1&2^2auxo.int^ vaccines. **(A)** Schematic of the construction of the tHIVconsvX immunogens (28). **(B)** The HIVconsv1 and HIVconsv2 (gray) synthetic sequences, were BCG codon optimized and fused to the 19-kDa lipoprotein signal sequence and inserted into the integrative p2auxo.HIVconsv1^int^ and p2auxo.HIVconsv2^int^ *E. coli*-mycobacterial shuttle plasmids, respectively. These shuttle vectors contain P α-Ag (in red), *Mycobacterium tuberculosis* α-antigen promoter, P HSP60, heat shock protein 60 gene promoter. The *glyA* and *LysA* (in yellow) complementing genes function as an antibiotic-free selection system in the auxotrophic strains of *E. coli* M15Δ*glyA* and BCG ΔLys, respectively. At the bottom, the process of plasmid integration in the BCG genome, based on the integration system of the L5 phage, is shown. The recognition occurs between the attachment sites AttP and AttB of the plasmid and mycobacterial genome, respectively (Att: attachment sites, in violet). **(C)** PCR analysis of attR and attL DNA regions of BCG.HIVconsv1^2auxo.int^ (left) and BCG.HIVconsv2^2auxo.int^ (right) colonies. Lanes 2 and 10: BCG wild type; Lanes 3 and 11: BCG.HIVconsvX^2auxo.int^ clone1; Lanes 4 and 12: BCG.HIVconsvX^2auxo.int^ clone 2; Lanes 5 and 13: BCG.HIVconsvX^2auxo.int^ clone 3; Lanes 6 and 14: BCG.HIVconsvX^2auxo.int^ clone 4; Lanes 7 and 15: negative control, distilled water; and lanes 1,8,9 and 16: molecular weight marker. **(D)** Western blot of BCG.HIVconsv1^2auxo.int^ and BCG.HIVconsv2^2auxo.int^ cell lysates. Lane 1: BCG wild type (negative control); Lane 2: BCG.HIVconsv1^2auxo.int^ working vaccine stock; Lane 3: BCG.HIVconsv2^2auxo.int^ working vaccine stock; Lane 4: BCG.Ø^2auxo.int^; Lane 5: Molecular weight marker.

The HIVconsv1&2 immunogens were designed by utilizing functionally conserved regions across group M strains, and include epitopes identified in untreated patients with low viral loads, as well as incorporating mosaic immunogens to improve global HIV-1 coverage and response breadth. The regions were reshuffled to decrease induction of T cells recognizing potential junctional epitopes (28) (Figure 1A). The HIVconsv1&2 open-reading frames were fused at their 5' end to nucleotides encoding the *M. tuberculosis* 19-kDa lipoprotein signal sequence, to facilitate antigen secretion, and fusion of foreign antigens to mycobacterial surface lipoproteins, thus improving their immunogenicity (5, 30). The chimeric 19-kDa signal sequence-HIVconsv1&2 genes were inserted into the *E. coli*-mycobacterial shuttle plasmids under the control of the *Mtb* α-antigen promoter to generate p2auxo.HIVconsv1^2auxo.int^ and p2auxo.HIVconsv2^2auxo.int^ (Figure 1B). Both plasmids contain the *E. coli* origin of replication (*oriE*), attachment sites (*attP*), and the integrase (*int*) genes from the mycobacteriophage L5 (31), and integrate as a single copy into a BCG chromosome (Figure 1B). It also contains the wild-type glycine A-complementing gene (*glyA*) and lysine A-complementing gene (*lysA5*) for vector selection and maintenance in the *E. coli* and the auxotrophic BCG strain, respectively (20). The p2auxo.HIVconsv1^int^ and p2auxo.HIVconsv2^int^ were obtained as previously described (23) and transformed into the glycine auxotrophic *E. coli* M15 (Δ*glyA*) strain and the lysine auxotrophic BCG Pasteur strain (Δ*lysA5*) (32, 33). The positive recombinant *E. coli* colonies were selected through culture on Minimal M9-D agar plates and the BCG.HIVconsv1&2^2auxo.int^ colonies on Middlebrook agar 7H10 medium without lysine supplementation. Integration of the p2auxo.HIVconsv1^int^ and p2auxo.HIVconsv2^int^ plasmid DNA into the mycobacterial genome was assessed by PCR analysis of attR and attL DNA regions. The BCG.HIVconsv1^2auxo.int^ or BCG.HIVconsv2^2auxo.int^ colonies were used as templates and bands of 766 and 874 bp corresponding to the attR and attL DNA regions, were detected (Figure 1C). Expression of the full size chimeric 19-kDa signal sequence-HIVconsv1 and HIVconsv2 proteins was confirmed by western blot analysis of BCG.HIVconsv1^2auxo.int^ and BCG.HIVconsv2^2auxo.int^ lysates (Figure 1D). The selected clones were preserved using the seed-lot system; master seed stocks and working vaccine stocks were prepared for further molecular characterization, immunogenicity and safety testing in mice.

### Genetic Identification and Characterization of BCG.HIVconsv1^2auxo.int^ and BCG.HIVconsv2^2auxo.int^

**Figure 2:**
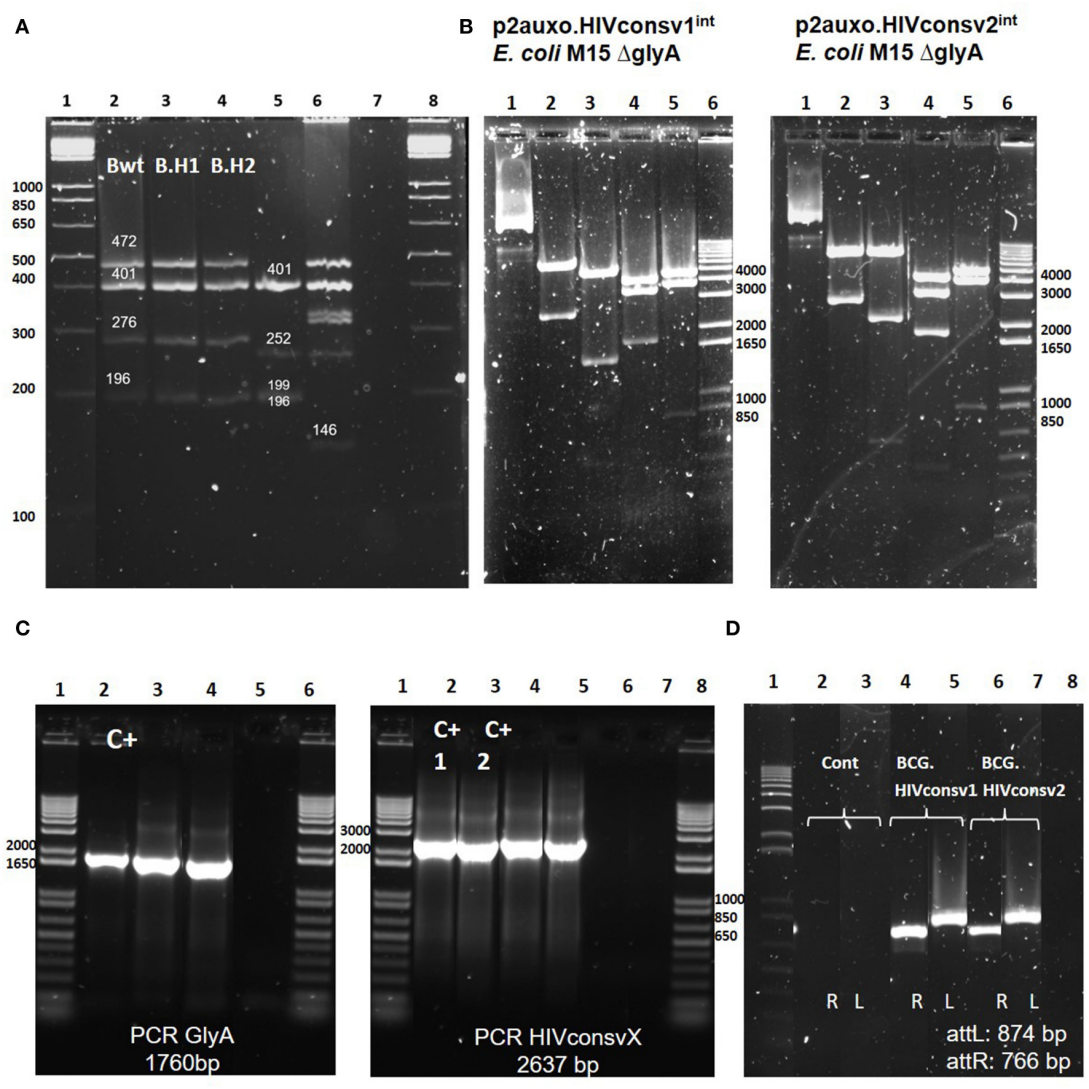
Genetic characterization of the BCG.HIVconsv1&2^2auxo.int^strains. **(A)** Pasteur substrain identification of BCG.HIVconsv1&2^2auxo.int^strains by multiplex PCR assay. Lane 1 and 8: molecular weight marker; lane 2: BCG wild type Pasteur giving the bands of 472, 401, 276, and 196 bp; lane 3 and 4: BCG.HIVconsv1&2^2auxo.int^ Working Vaccine Stocks (WVS), respectively, giving the bands of 472, 401, 276, and 196 bp; lane 5: BCG Connaught giving the bands of 401, 252,199, and 196 bp; lane 6: MTBVAC.HIVA^2auxo.int^ (*M.tuberculosis* strain) WVS giving a distinctive band of 146 bp, and lane 7: negative control, distilled water. **(B)** Enzymatic restriction analysis of p2auxo.HIVconsv1^int^ (left) and p2auxo.HIVconsv2^int^ (right) plasmids DNA purified from *E. coli* M15Δ*glyA* cultures (pre-BCG transformation). Lane 1: Undigested plasmids; Lane 2: HindIII digestion; Lane 3: PstI digestion; Lane 4: StuI digestion; Lane 5: XbaI digestion; Lane 6: Molecular weight marker. **(C)** PCR analysis of *E. coli glyA* DNA and HIVconsv1 or HIVconsv2 DNA coding sequences. Left panel: *glyA* PCR. Lanes 1 and 6: Molecular weight marker; Lane 2: p2auxo.Ag85B, positive control; Lane 3: BCG.HIVconsv1^int^ (WVS); Lane 4: BCG.HIVconsv2^int^ (WVS); Lane 5: distilled water, negative control. Right panel: HIVconsv1 and HIVconsv2 PCR. Lanes 1 and 8 corresponds to the molecular weight marker; Lanes 2 and 3: p2auxo.HIVconsv1 and p2auxo.HIVconsv2 (episomal), positive controls; Lane 4: BCG.HIVconsv1^int^ (WVS); Lane 5: BCG.HIVconsv2^int^ (WVS); Lanes 6 and 7: distilled water, negative controls; **(D)** PCR analysis of attR and attL DNA region using as template the cultures of BCG wild type, negative control (lane2 and 3), BCG.HIVconsv1^2auxo.int^ WVS (lane 4 and 5), BCG.HIVconsv2^2auxo.int^ WVS (lanes 6, and 7) and distilled water as negative control (lane 8). Lane 1 corresponds to molecular weight marker.

BCG substrains have been freeze-dried since the 1960s to maintain characteristics of the primary seed lots. Genetic identification techniques allow differentiation of the BCG substrains. To confirm that the molecular profiles of the BCG.HIVconsv1&2^2auxo.int^ vaccine strains corresponded to the BCG Pasteur substrain, we performed a multiplex PCR based method targeting SenX3-RegX3 regions and the BCG deletion regions including RD1, 2, 8, 14, and 16 (29). This method allows differentiation of substrains, and 196-bp products were generated using the primers ET1–3, indicating deletions of the RD1 region. In addition, in the BCG Pasteur substrain (BCG.HIVconsv1&2^2auxo.int^), the RD8 and RD16 were present, and generated products of 472 and 401 bp, respectively. The primers for the SenX3-RegX3 region generated products of 276 bp in BCG Pasteur. The PCR fingerprints of BCG Pasteur and BCG Connaught substrains (Figure 2A) were consistent with previously published results on genetic information of BCG substrains (29). In addition, the PCR pattern of MTBVAC.HIVA (34), a live attenuated *M. tuberculosis* strain expressing the HIVA immunogen (22), was consistent with the expected *M. tuberculosis* pattern.

To perform molecular characterization of the p2auxo.HIVconsv1^int^ and p2auxo.HIVconsv2^int^ plasmid DNA, enzymatic restriction and PCR analysis were performed. The plasmid DNA was purified from the *E. coli* M15 Δ*glyA* strain, and the enzymatic restriction pattern obtained displayed the same enzymatic pattern as compared with the predicted pattern of the plasmid DNA sequence (Figure 2B): HindIII digestion (Lane 2; bands of 6867 and 2637 bp), PstI digestion (Lane 3; bands of 5583, 1518, 537, and 315 bp), StuI digestion (lane 4; bands of 4224, 3460, and 1820 bp), XbaI digestion (Lane 5; bands of 4771, 3905 and 829 bp). Next, we performed PCR analysis using specific primers for the HIVconsv1, HIVconsv2, and *E. coli glyA* DNA coding sequences using the BCG.HIVconsv1^2auxo.int^ or BCG.HIVconsv2^2auxo.int^ Working Vaccine Stocks as template. Bands of 1760 and 2637 bp, corresponding to the size of the *E. coli glyA* DNA sequence and the HIVconsv1&2 sequences were detected (Figure 2C). Furthermore, PCR analysis using specific primers was employed to confirm integration of the p2auxo.HIVconsvX^int^ plasmid DNA into the parental BCG Δ*lysA* strain genome. The BCG.HIVconsv1^2auxo.int^ or BCG.HIVconsv2^2auxo.int^ Working Vaccine Stocks were used as template. Bands of 766 and 874 bp corresponding to the respective attR and attL attachment sites were detected in Working Vaccine Stocks, but not in BCG wt (Figure 2D).

### Phenotypic Characterization of the BCG.HIVconsv1^2auxo.int^ and BCG.HIVconsv2^2auxo.int^

**Figure 3:**
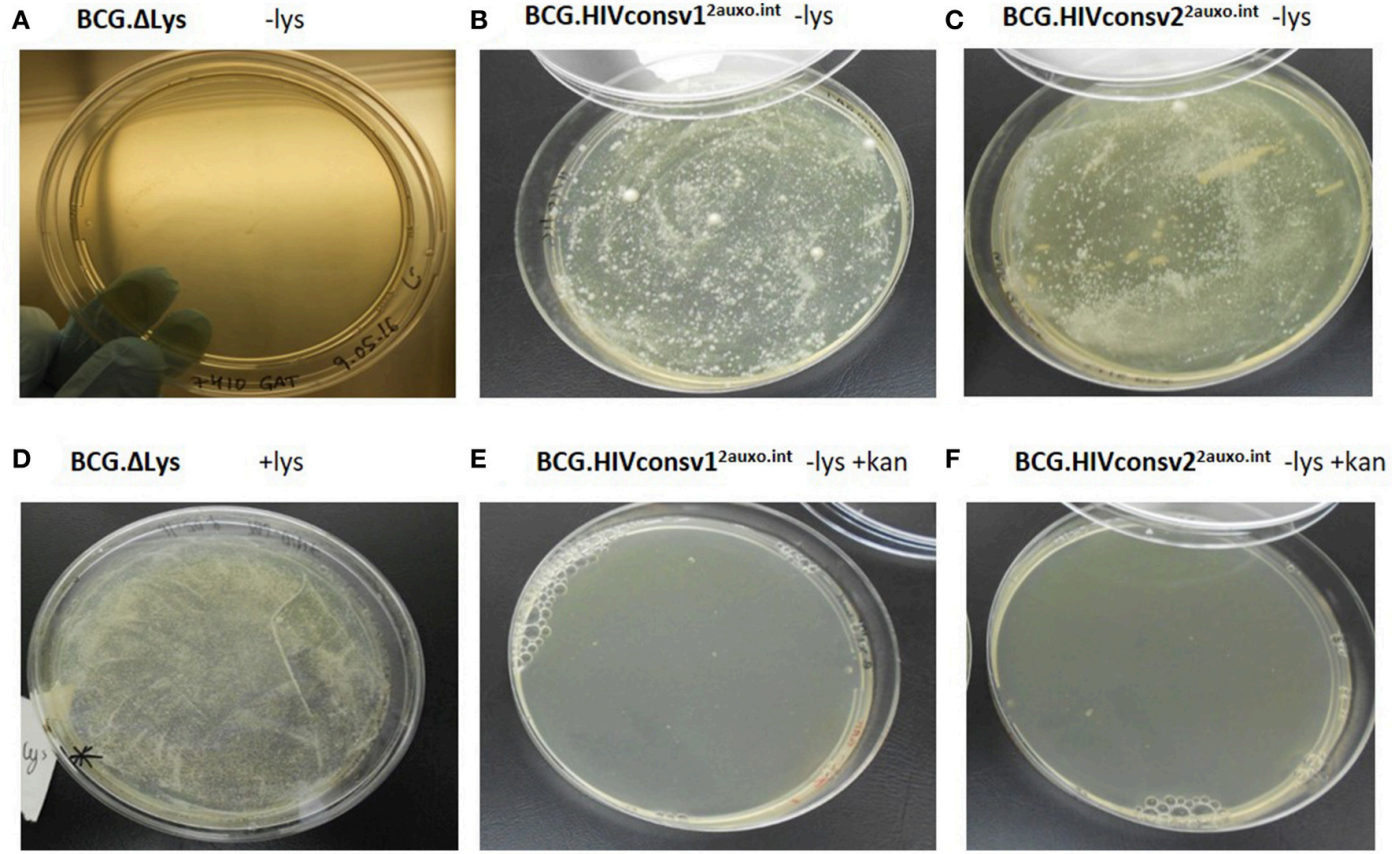
Phenotypic characterization of the BCG.HIVconsv1&2^2auxo.int^ vaccine strains. We assessed the phenotype of lysine auxotrophy, lysine complementation and kanamycin resistance. BCG lysine auxotroph, BCG.HIVconsv1^2auxo.int^ WVS and BCG.HIVconsv2^2auxo.int^ WVS strains plated on non-lysine supplemented 7H10 [**(A–C)**, respectively]; BCG lysine auxotroph strain plated on lysine supplemented 7H10 **(D)**; BCG.HIVconsv1^2auxo.int^ WVS and BCG.HIVconsv2^2auxo.int^ WVS strains [**(E**,**F)**, respectively] plated on 7H10 without lysine supplementation and with kanamycin.

To prevent plasmid instability, both *in vivo* and *in vitro*, as well as potential genetic rearrangements, several factors should be considered when constructing mycobacterium-based vaccine candidates. We previously demonstrated that the use of weak promoters (*Mycobacteria spp*. α-antigen promoter) and use of the BCG lysine auxotrophy-complementation system prevent disruption of gene expression due to genetic rearrangements (23, 35). The lysine auxotrophic BCG strain was used in combination with lysine gene complementation as an antibiotic-free plasmid selection and maintenance system. To phenotypically assess the stability of this system, the lysine auxotrophic BCG strain was cultured on lysine-supplemented and non-supplemented agar. The strain failed to grow on non-lysine supplemented (Figure 3A), but did, however, grow on agar plates supplemented with lysine (Figure 3D). In line with previous findings, the untransformed lysine auxotrophic BCG strain failed to grow without the presence of lysine, and lysine complementation provided by the p2auxo plasmid in the BCG.HIVconsv1&2^2auxo.int^ strains abolished the requirement for exogenous lysine (Figures 3B,C). As expected, the lysine auxotrophic strain did grow upon supplementation with lysine. Furthermore, attempts to culture these strains on agar plates containing kanamycin resulted in no growth (Figures 3E,F), confirming the lack of kanamycin resistance.

### The BCG.HIVconsv1^2auxo.int^ and BCG.HIVconsv2^2auxo.int^ Vaccines Are Genetically Stable

**Figure 4:**
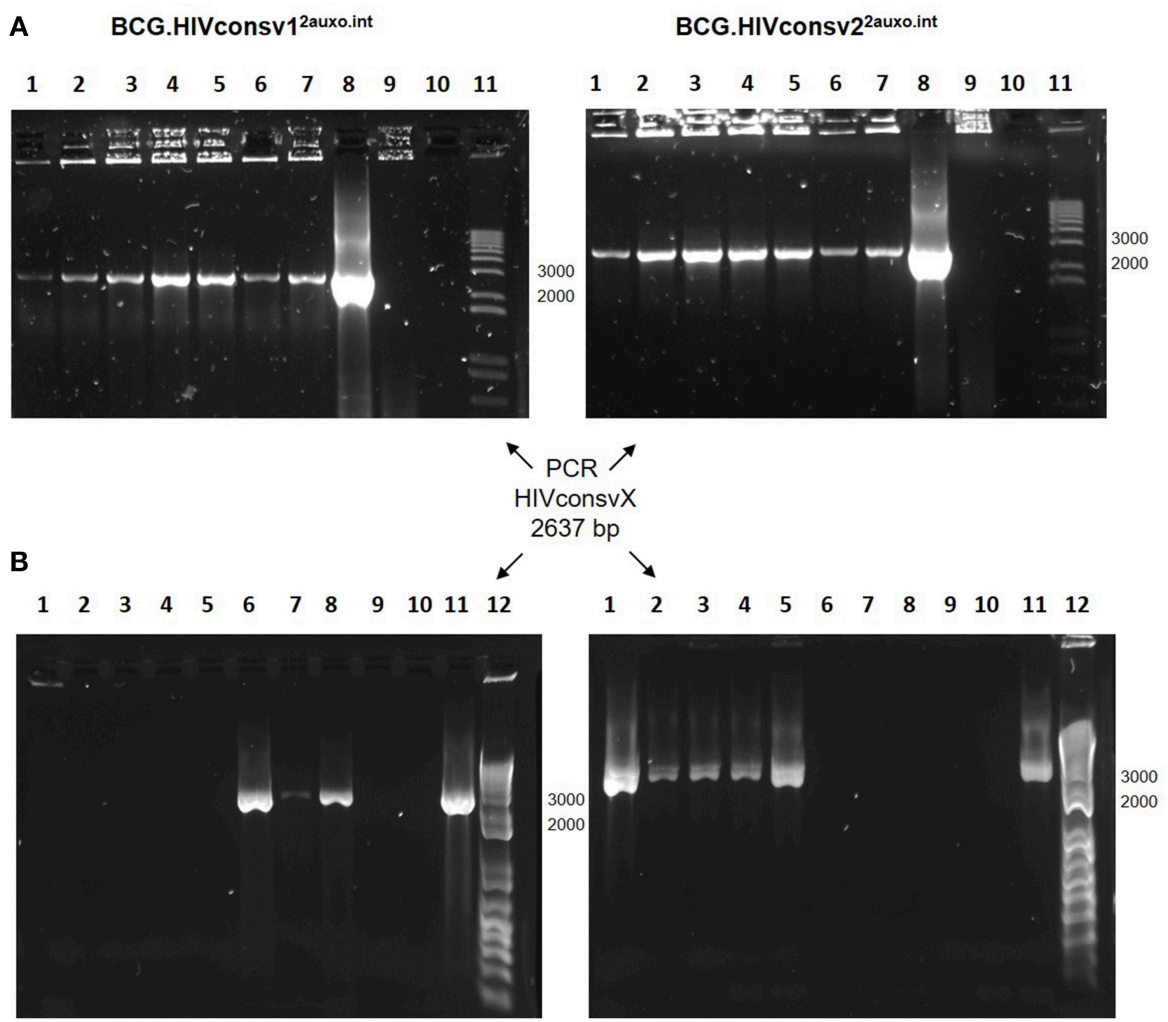
Genetic *s*tability of the BCG.HIVconsv1^2auxo.int^ and BCG.HIVconsv2^2auxo.int^ strains. **(A)** *In vitro* stability. Working vaccine stocks were subcultured in lysine-free medium for 5 weeks and PCR analysis was to confirm presence of the HIVconsv1&2 genes; BCG.HIVconsv1^2auxo.int^ (left panel) and BCG.HIVconsv2^2auxo.int^ (right panel). Lanes 1–5 correspond to weekly subcultures from week 1–5; Lanes 6&7: 1 and 2 weekly subcultures in lysine supplemented media; Lanes 8: p2auxo.HIVconsvX^int^ plasmid (positive control); Lanes 9: distilled water (negative control); Lanes 10: BCG wt (negative control); Lanes 11: molecular weight marker. The HIVconsv1&2 genes were detected in both BCG.HIVconsv1^2auxo.int^ and BCG.HIVconsv2^2auxo.int^ strains after 5 weekly subcultures. **(B)** *In vivo* stability. Mice were injected i.d. with 10^6^ cfu of BCG.HIVconsv1&2^2auxo.int^ and boosted i.m. with 10^8^ IU of ChAdOx1.HIVconsv5&6, spleens were homogenized 8 weeks after BCG inoculation and the recovered rBCG colonies were tested by PCR analysis using primers specific for HIVconsv1 (left panel) or HIVconsv2 (right panel) DNA coding sequences. Representative assessment of positive colonies. Lanes 1 to 8: rBCG.HIVconsvX^2auxo.int^ colonies recovered from the non-lysine supplemented plate; Lanes 9: BCG wt (negative control); Lanes 10: Distilled water (negative control); Lanes 11: p2auxo.HIVconsv1^int^ or p2auxo.HIVconsv2^int^, respectively (Plasmid DNA positive control); Lanes 12: Molecular weight marker.

To evaluate the *in vitro* genetic stability of the p2auxo.HIVconsvX.int plasmid DNAs, working vaccine stocks were subcultured in lysine free medium and PCR analysis was performed using each subculture as template. The HIVconsvX genes were detected in both BCG.HIVconsv1^2auxo.int^ and BCG.HIVconsv2^2auxo.int^ strains after 5 weekly subcultures in selective medium without lysine supplementation, as well as two weekly subcultures in lysine supplemented medium (Figure 4A). Next, we wanted to assess genetic stability of the p2auxo.HIVconsvX.int *in vivo*, several weeks after immunization. For this, mice were immunized with 10^6^ CFU of a 1:1 mixture of BCG.HIVconsv1^2auxo.int^ and BCG.HIVconsv2^2auxo.int^ and after 5 weeks boosted with 5 × 10^7^ IU of ChAdOx1.tHIVconsv5 mixed with 5 × 10^7^ IU of ChAdOx1.tHIVconsv6. Two weeks later, spleens were harvested and homogenized for culture on 7H10 agar plates. Resulting BCG colonies were analyzed by PCR for presence of both HIVconsv1 and HIVconsv2 genes, eight out of eight colonies contained either the HIVconsv1 or HIVconsv2 gene, with 31% of retrieved colonies being positive for HIVconsv1 and 69% being positive for HIVconsv2 (Figure 4B).

### The BCG.HIVconsv1&2^2auxo.int^ Prime-ChAdOx1.tHIVconsv5&6 Boost Regimen Elicits HIV-1-Specific T-Cell Responses

**Figure 5:**
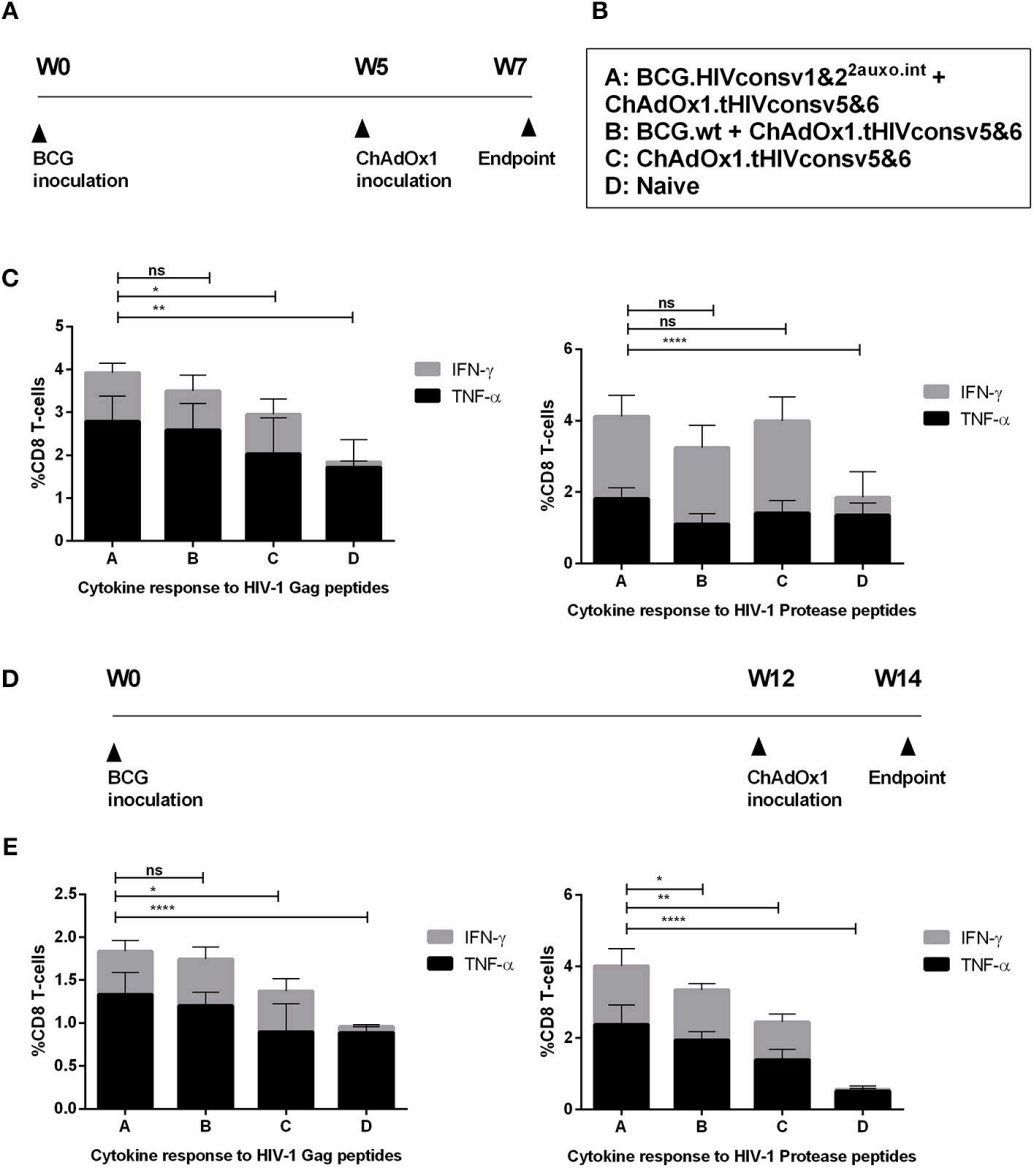
Induction of HIV-1-specific T-cell responses by the BCG.HIVconsv1&2^2auxo.int^ ChAdOx1.tHIVconsv5&6 prime-boost regimen in the BALB/c mice. Adult mice (7-weeks-old, *n* = 8/group) were either left unimmunized or primed with 10^6^ cfu of BCG.HIVconsv1&2^2auxo.int^ or BCG wild type (intradermally) and boosted with 10^8^ IU of ChadOx1.tHIVconsv5&6 (intramuscularly) 5 weeks post-BCG inoculation. Mice were sacrificed 2 weeks later for T-cell analysis. The immunization schedule is depicted in **(A)** and the groups in **(B)**. **(C)** Analysis of IFN-γ and TNF-α, vaccine elicited HIV-1-peptide specific CD8+ T-cell responses to the immunodominant Gag peptide epitopes (AMQMLKDTI and AMQMLKETI) and Prot peptides (VLVGPTPVNI and VLIGPTPVNI). The cumulative frequencies of CD8^+^ T-cells producing cytokines are shown. Data are presented as group means, error bars represent standard deviation. Statistics were performed by One-way ANOVA on the sums of IFN-γ and TNF-α producing CD8+ T-cells. **(D)** Adult mice (7-weeks-old, *n* = 8/group) were either left unimmunized or primed with 10^6^ CFU of BCG.HIVconsv1&2^2auxo.int^ or BCG wild type (intradermally) and boosted with 10^8^ IU of ChadOx1.tHIVconsv5&6 (intramuscularly) 12 weeks post-BCG inoculation. Mice were sacrificed 2 weeks later for T-cell analysis. **(E)** Analysis of IFN-γ and TNF-α vaccine elicited HIV-1- peptide specific CD8+ T-cell responses to the immunodominant Gag peptide epitopes (AMQMLKDTI and AMQMLKETI) and Prot peptides (VLVGPTPVNI and VLIGPTPVNI). The cumulative frequencies of CD8^+^ T-cells producing cytokines are shown. Data are presented as group means, error bars represent standard deviation. Statistics were performed by One-way ANOVA on the sums of IFN-γ and TNF-α producing CD8+ T-cells. *p < 0.05, **p < 0.005, ***p < 0.001, and ****p < 0.0001.

We previously demonstrated, that lysine auxotrophic BCG transformed with the p2auxo integrative plasmid expressing an HIV-1 immunogen is suitable for use in prime boost regimens, e.g., when combined with an MVA-vectored boost (23). In this study, we evaluated the specific HIV-1 T-cell responses in BALB/c mice after immunization with BCG.HIVconsv1&2^2auxo.int^ prime and ChAdOx1.tHIVconsv5&6 boost in order to assess the level of enhancement of immune responses provided by the recombinant BCG expressing the HIVconsv1&2 immunogens, compared to mice receiving BCGwt or ChAdOx1.tHIVconsv5&6 alone. The tHIVconsvX immunogens were designed to induce broad and robust T-cell responses, and include epitopes identified in untreated patients with low viral loads (28). The immunogenicity readout focused on the immunodominant H-2^d^ restricted CTL HIV-1 Gag peptide epitopes AMQMLKDTI and AMQMLKETI representing HIVconsv1 and HIVconsv2, respectively, as well as the HIV-1 protease (prot) peptides, VLVGPTPVNI and VLIGPTPVNI (26, 36–38). Adult mice were immunized with BCG.HIVconsv1&2^2auxo.int^ and after 5 weeks, the animals received a ChAdOx1.tHIVconsv5&6 boost (Figures 5A,B). Mice were sacrificed 2 weeks after the boost and the cytokine production capacity of CD8^+^ T-cells from vaccinated mice upon stimulation with the HIV-1 peptides was assayed by intracellular cytokine staining. A significantly higher frequency of TNF-α secreting CD8+ T-cells was observed in mice immunized with BCG.HIVconsv1&2^2auxo.int^ as compared to mice receiving only ChAdOx1.tHIVconsv5&6 in response to the AMQMLKD/ETI peptides (*p* = 0.0086). When comparing sums of CD8+ T-cells secreting IFN-γ and TNF-α, the BCG.HIVconsv1&2^2auxo.int^ primed mice, performed significantly better than mice not receiving BCG prime immunization (*p* = 0.0380). Differences in IFN-γ secretion alone were not significant between vaccinated groups. There were also no differences observed for individual cytokine parameters when compared to mice primed with BCGwt. In response to the VLV/IGPTPVNI peptides, there were no differences between any of the vaccination groups regarding the total cytokine secreting CD8^+^ T-cells (IFN-γ secreting CD8^+^ T-cells + TNF-α secreting CD8^+^ T-cells). However, frequencies of TNF-α were significantly higher in mice vaccinated with BCG.HIVconsv1&2^2auxo.int^ than in BCGwt receiving mice (Figure 5C; *p* = 0.0099).

BCG is a slow-growing organism which infects APCs and provides a persistent low-level antigenic exposure (39). For this reason, we were interested in evaluating the effects of a longer immunization schedule on not only the magnitude of immune responses, but also the differences in cytokine profiles, and possible shifts in response to the peptides, i.e., increased or decreased responses to the Gag peptides as compared to the Prot peptides. Thus, mice were immunized as previously described and boosted after 12 weeks instead of 5 weeks and sacrificed 2 weeks after the boost (Figure 5D). Significantly higher total cytokine production (sums of IFN-γ and TNF-α secreting CD8^+^ T-cells) were observed following BCG.HIVconsv1&2^2auxo.int^ prime as compared to mice receiving only ChAdOx1.tHIVconsv5&6 after stimulation with AMQMLKD/ETI peptides (*p* = 0.02). Specifically, the frequency of TNF-α secreting cells was enhanced (*p* < 0.0001). No significant difference was observed when compared with BCG wt primed mice. Interestingly, the enhancement of cytokine responses provided by BCG.HIVconsv1&2^2auxo.int^ to VLV/IGPTPVNI peptides was improved in the longer regimen as compared to the 7-week regimen. The frequencies of total cytokine secreting CD8^+^ T-cells was significantly higher in mice primed with BCG.HIVconsv1&2^2auxo.int^, as compared to BCG wt immunized mice, and mice receiving ChAdOx1.tHIVconsv5&6 alone (Figure 5E, *p* = 0.0408, *p* = 0.0025 and *p* < 0.0001, respectively). When comparing the individual parameters, IFN-γ secretion was significantly higher in mice primed with BCG.HIVconsv1&2^2auxo.int^ as compared to mice only receiving ChAdOx1.tHIVconsv5&6 (*p* = 0.0003), but no significant differences were observed between mice primed with BCGwt and BCG.HIVconsv1&2^2auxo.int^. On the other hand, frequencies of TNF-α secreting CD8+ T-cells were significantly higher in BCG.HIVconsv1&2^2auxo.int^ primed mice than in both BCGwt primed and the group receiving ChAdOx1.tHIVconsv5&6 alone (*p* = 0.0130 and *p* < 0.0001, respectively). Similar results were observed when evaluating BCG.HIVconsv1&2^2auxo.int^ in combination with MVA.tHIVconsv3&4 (Figure S1A). IFN-γ secretion in response to 10 peptide pools spanning the HIVconsvX proteome (40) was assessed by ELISpot, and CD8^+^ T-cell and CD4^+^ T-cell cytokine secretion in response to three peptide pools was assessed by intracellular cytokine staining. The total IFN-γ secretion as assessed by ELISpot in response to all peptide pools was significantly higher than in mice receiving rMVA alone. However, only a trend toward higher secretion was observed when compared to BCGwt primed mice. No statistically significant differences were observed between BCGwt primed mice and mice not receiving any prime (Figure S1B). Cytokine production in CD8^+^ and CD4^+^ T-cells in response to the three peptide pools (pool 1 containing the AMQMLKD/ETI and pool 4 containing the VLV/IGPTPVNI peptides) revealed similar trends toward higher total cytokine secretion as observed in the BCG.HIVconsv1&2 + ChAdOx1.tHIVconsv5&6 immunization regimen, although no statistically significant differences were observed between groups (Figures S1C,D).

### The BCG.HIVconsv1&2^2auxo.int^ ChAdOx1.tHIVconsv5&6 Regimen Boost Is Well Tolerated

**Figure 6:**
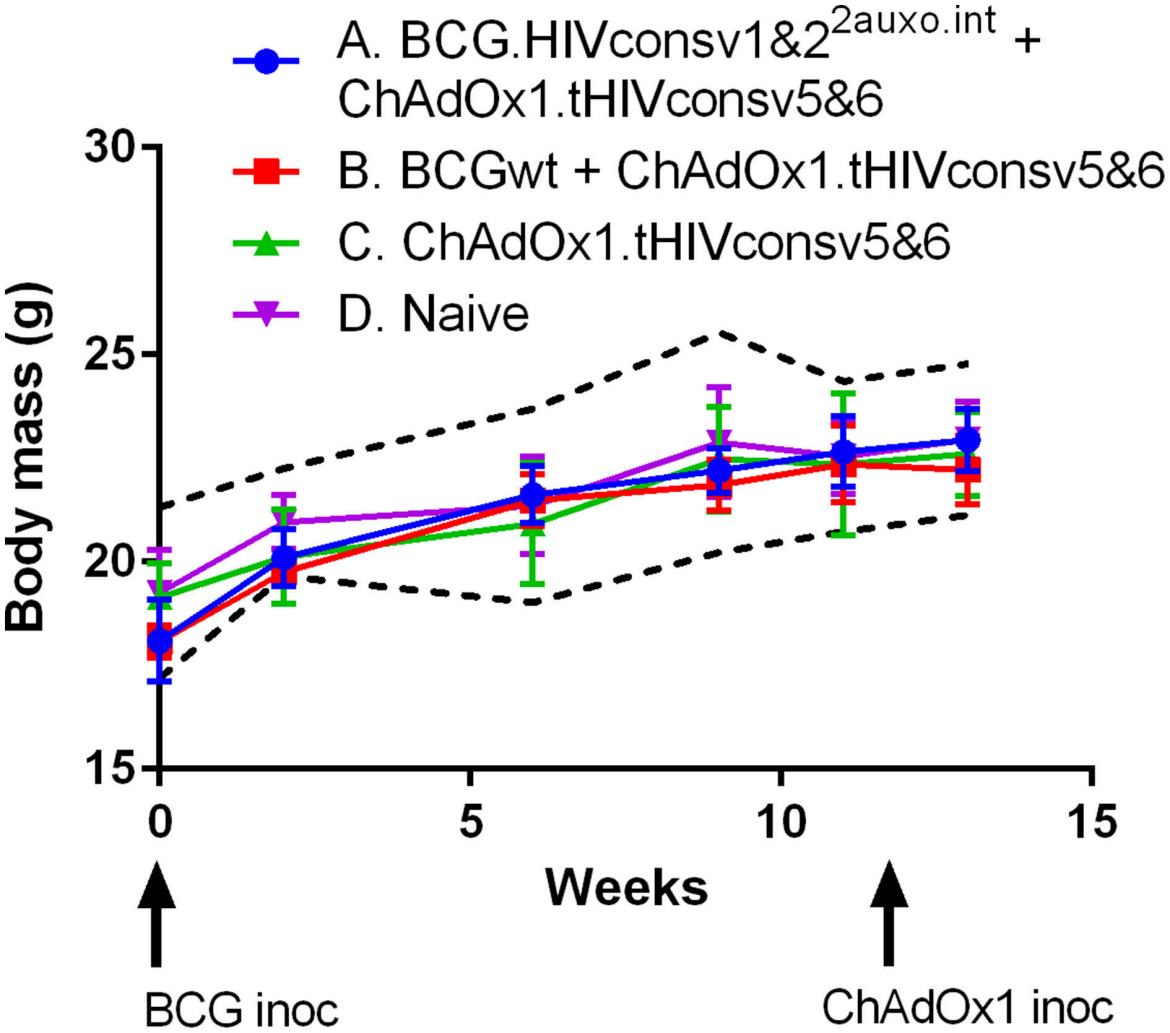
Safety of BCG.HIVconsv1&2^2auxo.int^ in mice. Mice were immunized i.d. with 10^6^ CFU of BCG.HIVconsv1&2^2auxo.int^ and boosted with 10^8^ IU of ChAdOx1.tHIVconsv5&6, body weights were recorded regularly, and the mean for each group of mice is shown (*n* = 5). Data from naive mice are presented as mean ± 2 SD (dashed black lines, *n* = 5).

Five adult mice per group were either left unimmunized or received 10^6^ colony forming units (cfu) of BCG wt or a total of 10^6^ cfu of BCG.HIVconsv1&2^2auxo.int^ intradermally and subsequently received boost of a total of 10^8^ IU of ChAdOx1.tHIVconsv5&6 and their body mass was monitored regularly over time (Figure 6). To carry out a rigorous safety assessment, the dose inoculated in mice was 10-fold higher than the usual dose given to adult mice following the guidance of the European Pharmacopeia for the safety testing of live vaccines. No statistically significant difference was observed between the vaccinated mice and the naïve mice at specific time points, corresponding to the time of BCG inoculation as well as 3 and 10 weeks after. The body mass profile was similar to the mouse provider's standard body mass curve (www.envigo.com). Furthermore, between weeks 0 and 10, the body mass monitored in all vaccinated mice was found between the mean ± 2 standard deviations (SD) body mass curve in naïve mice (Figure 6). No vaccine-related deaths, no local adverse events, and no associated systemic reactions were observed.

## Discussion

Several prevention strategies for HIV-1 transmission have been explored and implemented throughout the years. In spite of this, 1.8 million new infections occurred in 2017 (41). The development of an effective, safe and affordable vaccine against HIV-1 remains the best option for halting the HIV-1/AIDS pandemic. In this study, we have constructed rBCG vaccine candidates expressing the second generation conserved-region T-cell immunogens, HIVconsv1 and HIVconsv2 (28), using the integrative antibiotic-resistance free *E. coli*-mycobacterial shuttle vector, p2auxo.int. We previously demonstrated that the *in vitro* stability of the integrative plasmid p2auxo.int expressing the HIV-1 subtype A derived immunogen, HIVA, was increased 4-fold as compared to the episomal plasmid in BCG in the absence of selective pressure (23). Here, we produced the BCG.HIVconsv1&2^2auxo.int^ vaccine candidates under Good Laboratory Practices (GLP)-compatible conditions, and characterized them genotypically and phenotypically, confirming presence of the HIVconsv1&2 genes and their products in lysates of working vaccine stocks. Moreover, we demonstrated that the BCG.HIVconsv1&2^2auxo.int^ vaccines in combination with ChAdOx1.tHIVconsv5&6 induced HIV-1-specific interferon-γ-producing T-cell responses in adult BALB/c mice and were well tolerated. This vaccine concept may well be worthy of further exploration and development in the pursuit of an effective HIV-1 vaccine, while simultaneously providing cellular immunity to *M. tb*, as we have demonstrated for multiple rBCG:HIV vaccine candidates (19, 20, 23, 35, 42).

Although antibiotic resistance is routinely employed for selection and maintenance of recombinant plasmids in *E. coli*, its use is not suitable for clinical trials (43, 44). We previously described the development of a new mycobacterial vaccine design using an antibiotic-free expression system for selection and maintenance for HIV vaccine development (23). In this study, we utilized the integrative *E. coli*-mycobacterial shuttle plasmid p2auxo.int, to express HIV-1 immunogens HIVconsv1&2. The vector contains a glycine complementing gene for *E. coli* and a lysine complementing gene for mycobacteria to allow selection and maintenance without the use of antibiotic resistance. Thorough characterization and monitoring of genetic stability in BCG-vectored HIV-1 vaccines is crucial to assure consistent quality of vaccine lots and induction of HIV-1-specific immune responses (45). The use of integrative vectors and auxotrophic complementation was shown to increase genetic stability of rBCG:HIV vaccines (46). Indeed, we demonstrate that the HIVconsv1&2 genes are stable in BCG both *in vitro* and *in vivo* after several bacterial passages. Genetic instability as well as procedures for rBCG preparation and handling can have detrimental effects on the immunopotency of vaccines (47, 48). BCG substrains can be identified using molecular methods. In this study, we performed a multiplex PCR assay to genetically determine the strain of the BCG.HIVconsv1&2^2auxo.int^ vaccine candidates and confirmed that the strain corresponded to the BCG Pasteur substrain.

It was demonstrated in both mouse and non-human primate studies that rBCG elicited HIV-1- and SIV-specific cell-mediated responses (45, 49–53). Several types of boosting vaccinations were evaluated in heterologous prime-boost regimens in combination with rBCG:HIV delivered as a priming vaccine. These studies showed rBCG to be a suitable priming vector, providing enhancement of specific T-cell responses through a heterologous boost (20, 52, 54, 55). In this study, the tHIVconsvX boosting vaccination was vectored by simian adenovirus of chimpanzee origin ChAdOx1, a vector which has the advantage of overcoming the detrimental effects of pre-existing anti-human adenovirus immunity. The chimpanzee adenoviral vector was safe and induced antigen-specific cellular and humoral immune responses in humans (56). During the course of this study, mice were regularly monitored for signs of malaise, along with their body mass. Importantly, no adverse effects were observed in any animals, and the body mass curves of mice receiving the rBCG-rChAdOx1 prime-boost regimen were similar to those of naïve mice.

Regarding the induction of HIV-1 specific immune responses, our readout focused on immunodominant peptide epitopes from HIV-1 Gag and Prot, respectively. We employed both a short immunization schedule of 7 weeks and a longer immunization schedule of 14 weeks, in both cases animals were sacrificed 2 weeks following the boost immunization. A group receiving BCGwt as a prime was included to allow comparison of the unspecific adjuvanticity that BCG immunization confers. In the short immunization schedule, the highest frequency of CD8^+^ T-cells producing cytokines in response to Gag derived peptides were seen in mice primed with BCG.HIVconsv1&2^2auxo.int^, these responses were statistically significantly higher than that observed for mice that did not receive any priming immunization. Although only a trend toward higher frequencies was observed in comparison with BCGwt-primed mice. Priming with the recombinant BCG did not enhance cytokine secretion in response to the Prot peptides. In the longer immunization schedule, BCG.HIVconsv1&2^2auxoint^ primed mice again had the highest total frequencies of CD8^+^ T-cells producing cytokines, when stimulated with Gag-derived peptides. However, only statistical significance was observed when comparing with mice receiving no BCG prime. Regarding the Prot-derived peptides, the priming effect of BCG.HIVconsv1&2^2auxoint^ immunized mice was improved in the longer regimen as compared to the 7-week regimen. Total cytokine secretion was significantly higher in mice primed with BCG.HIVconsv1&2^2auxo.int^, as compared to BCG wt immunized mice, mice receiving ChAdOx1.tHIVconsv5&6 alone, as well as to naïve mice. When comparing individual cytokine parameters, the differences were largely dependent on TNF-α, but IFN-γ was significantly increased compared to mice not receiving any BCG prime immunization. Both IFN-γ and TNF-α have been shown to be important in inducing both CD4^+^ and CD8^+^ CTLs in the context of mycobacteria (57). Furthermore, anti-TNF-α treatment has been shown to reduce perforin and granulysin expression in lymphocytes. *M. tuberculosis*-specific CD8^+^CCR7^−^CD45RA^+^ effector memory T cells have also been shown to express TNF-α on the cell surface, and anti-TNF-α treatment was associated with a reduction of this T-cell subset as well as decreased antimicrobial activity against *M. tuberculosis* (58).

Similar trends toward higher total cytokine secretion were observed when the BCG.HIVconsv1&2 was delivered in a 6-week prime-boost regimen in combination with MVA.tHIVconsv5&6, although no statistically significant differences were observed between groups (Figure S1).

The limitations of the study include the fact that the vaccine combination was assessed in immunocompetent hosts, and a model such as the SCID mouse model, would be more suitable for evaluation and confirmation of safety in an immunocompromised host. In addition, the assessment of different routes, doses, and immunization schedules would provide better knowledge toward the optimal vaccination regimen. Finally, the readout of this study focused on cytokine production in response to two immunodominant peptide variants, further information regarding specific T-cell subsets and more peptides would provide a more complete picture of the immune response at hand.

In this study, we constructed, characterized and pre-clinically evaluated novel, safe, good laboratory practice–compatible BCG-vectored HIV-1 vaccine candidates expressing the HIVconsv1 and HIVconsv2 immunogens. In conclusion, when comparing total cytokine secretion, these antibiotic-resistance free BCG.HIVconsv1&2 vaccine candidates boosted specific responses to the second generation tHIVconsvX proteins following a boosting vaccination vectored by ChAdOx1. While further studies such as assessments of other parental strains, doses, and immunization routes should be performed before establishing its applicability as a vaccine platform, this conserved vaccine concept consisting of a rBCG-ChAdOx1 prime-boost regimen targeting HIV-1 genetic variability may be a useful tool in the fight against HIV-1/AIDS.

## Ethics Statement

Immunological mice experiments were approved by the local Research Ethics Committee (Procedure Med 365/16, Clinical Medicine, School of Medicine and University of Barcelona) and by the Ethical Committee for animal experimentation from University of Barcelona and strictly conformed to Generalitat de Catalunya animal welfare legislation.

## Author Contributions

JJ and TH conceived and planned the experiments and study design. AK, NG, and KR performed the experimental work with support and under supervision of NS, TH, and JJ. NS contributed to the interpretation of the results. AK took the lead in writing the manuscript. NM, EW, NS, JJ, and TH provided critical feedback and helped shape the research, analysis, and manuscript. JJ took the lead in revising the manuscript.

### Conflict of Interest Statement

TH is co-inventor on the Mosaic Conserved Region HIV Immunogenic Polypeptides patents PCT/US2014/058422 and EP14846993.5. The remaining authors declare that the research was conducted in the absence of any commercial or financial relationships that could be construed as a potential conflict of interest.

## Supplementary Material

Figure S1 Induction of HIV-1 specific T-cell responses BCG.HIVconsv1&2^2auxo.int^ + MVA.tHIVconsv3&4 in immunized BALB/c mice. Adult mice (6-weeks-old, *n* = 5/group) were immunized with either 10^6^ cfu of BCG.HIVconsv1&2^2auxo.int^ (id) and boosted with 5 × 10^6^ PFU MVA.tHIVconsv3&4 (im) after 5 weeks (group A), or with 10^6^ BCG.wt (id) and boosted with 5 × 10^6^ PFU MVA.tHIVconsv3&4 (im) after 5 weeks (group B), or not primed with BCG but immunized with 5 × 10^6^ PFU MVA.tHIVconsv3&4 (im) at week 5 (group C), or immunized only with BCG.HIVconsv1&2^2auxo.int^ at week 1 (group D). Mice were sacrificed 1 week later for analysis of T-cell responses **(A)**. Splenocytes of the vaccinated mice were stimulated with 10 peptide pools of the HIVconsv immunogens and IFN-γ ELISpot was performed, a sum of IFN-γ SFC/10^6^ in response to the 10 peptide pools (40) was calculated for each mouse. The graphs represent the mean response per group and the bars standard deviation. Statistics were performed using Mann-Whitney tests with the Graphpad Prism 6.0 software **(B)**. Analysis of IFN-γ, IL-2, and TNF-α vaccine elicited HIV-1-peptide pool specific T-cell responses. Splenocytes of vaccinated mice were stimulated with the three most reactive peptide pools and intracellular cytokine staining was performed to assess CD8^+^ T-cell responses **(C)** and CD4^+^ T-cell responses **(D)**.
