# Supplementary figures and images for: Priming With Recombinant BCG Expressing Novel HIV-1 Conserved Mosaic Immunogens and Boosting With Recombinant ChAdOx1 Is Safe, Stable, and Elicits HIV-1-Specific T-Cell Responses in BALB/c Mice
Source: Front Immunol. 2019 May 14;10:923. doi: 10.3389/fimmu.2019.00923 (PMC6530512; doi:10.3389/fimmu.2019.00923)

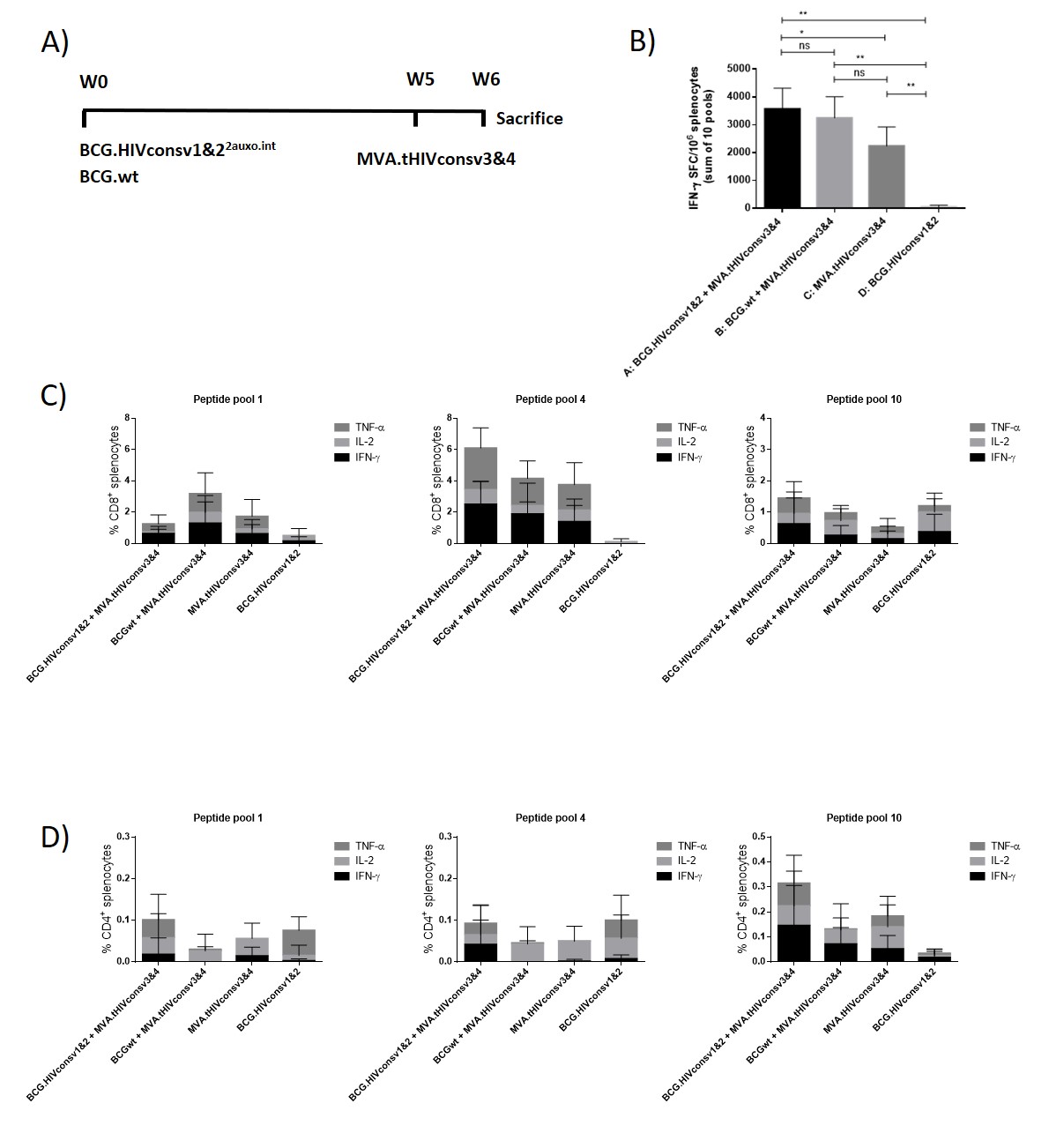

Supplement: Figure S1 — Induction of HIV-1 specific T-cell responses BCG.HIVconsv1&22auxo.int + MVA.tHIVconsv3&4 in immunized BALB/c mice. Adult mice (6-weeks-old, n = 5/group) were immunized with either 106 cfu of BCG.HIVconsv1&22auxo.int (id) and boosted with 5 × 10^6^ PFU MVA.tHIVconsv3&4 (im) after 5 weeks (group A), or with 106 BCG.wt (id) and boosted with 5 × 10^6^ PFU MVA.tHIVconsv3&4 (im) after 5 weeks (group B), or not primed with BCG but immunized with 5 × 10^6^ PFU MVA.tHIVconsv3&4 (im) at week 5 (group C), or immunized only with BCG.HIVconsv1&22auxo.int at week 1 (group D). Mice were sacrificed 1 week later for analysis of T-cell responses (A). Splenocytes of the vaccinated mice were stimulated with 10 peptide pools of the HIVconsv immunogens and IFN-γ ELISpot was performed, a sum of IFN-γ SFC/106 in response to the 10 peptide pools (40) was calculated for each mouse. The graphs represent the mean response per group and the bars standard deviation. Statistics were performed using Mann-Whitney tests with the Graphpad Prism 6.0 software (B). Analysis of IFN-γ, IL-2, and TNF-α vaccine elicited HIV-1-peptide pool specific T-cell responses. Splenocytes of vaccinated mice were stimulated with the three most reactive peptide pools and intracellular cytokine staining was performed to assess CD8+ T-cell responses (C) and CD4+ T-cell responses (D). [file Image_1.JPEG]
